# Complete genome sequencing of three methicillin-resistant *Staphylococcus aureus* isolated from the blood of patients with persistent bacteremia

**DOI:** 10.1128/mra.01177-23

**Published:** 2024-07-11

**Authors:** Soo In Lee, Seongman Bae, Yang Soo Kim, Sungkyoung Lee

**Affiliations:** 1 Department of Bacterial Disease Research, National Institute of Health, Korea Disease Control and Prevention Agency, Cheongju, Republic of Korea; 2 Division of Infectious Diseases, Department of Internal Medicine, Asan Medical Center, University of Ulsan College of Medicine, Seoul, Republic of Korea; University of Maryland School of Medicine, Baltimore, Maryland, USA

**Keywords:** MRSA, bacteremia, genome sequencing

## Abstract

We report the complete genome sequences of three molecular types of methicillin-resistant *Staphylococcus aureus* (MRSA) clinical strains isolated from the blood of three patients diagnosed with persistent MRSA bacteremia: KNIH_5618 (ST5-t5076-SCC*mec*II), KNIH_5844 (ST72-t664-SCC*mec*IV), and KNIH_6268 (ST89-t375-SCC*mec*II). These genome sequences contribute to an enhanced understanding of the underlying causes of persistent MRSA infection.

## ANNOUNCEMENT

Methicillin-resistant *Staphylococcus aureus* (MRSA) causes bloodstream infections and thus poses a significant threat to public health worldwide (1). In particular, persistent MRSA bacteremia is a global healthcare concern and is associated with poor treatment outcomes (2, 3). In this study, we report the complete genome sequence of three MRSA isolates, KNIH_5618, KNIH_5844, and KNIH_6268, which caused bloodstream infections that persisted for at least 14 days despite antibiotic therapy. The KNIH_5618, KNIH_5844, and KNIH_6268 were obtained on 26 October 2018, 27 May 2019, and 1 December 2020, respectively, from the initial blood cultures of patients with persistent *S. aureus* bacteremia who were admitted to the Asan Medical Center in Korea. All isolates were identified as oxacillin-resistant and vancomycin-susceptible *S. aureus* strains by determining the minimum inhibitory concentration according to the Clinical and Laboratory Standard Institute guidelines (4). They were subcultured on the blood agar plates (BAPs) and stored in tryptic soy broth (TSB) with 15% glycerol at −70°C. Stock solutions of the isolates were streaked onto BAPs and incubated at 37°C for 20 h to obtain single colonies. A single colony was then inoculated in TSB and grown overnight at 37°C with shaking at 200 rpm. Genomic DNA was extracted using DNeasy Blood & Tissue kit (Qiagen, Hilden, Germany) supplemented with lysostaphin (200 µg/mL), following the manufacturer’s instructions. Purified genomic DNA was sheared and fragmented to a size of 10 kb using a Covaris G-tube (Covaris, Woburn, MA, USA). High-fidelity (HiFi) sequencing libraries were generated using the SMRTbell Prep Kit 3.0 (PacBio, Menlo Park, CA, USA) and purified using AMPure PB beads (PacBio, Menlo Park, CA, USA). Genome sequences were obtained using the PacBio Sequel IIe system (PacBio, Menlo Park, CA, USA). The quality of sequence reads was examined using NGSQCToolkit version 2.3.3 (5), *de novo* assembly was performed using SMRT Link version 12.0.0.177059 (6), and the quality of the assembled genomes was assessed using BUSCO version 5.4.5 (7). Default parameters were used except where otherwise noted. The assembled sequences were annotated using the NCBI Prokaryotic Genome Annotation Pipeline version 6.5 (8). The annotation results were input into Proksee version 1.1.2 to generate circular maps for the complete genomes. The assembled genome sizes ranged from 2,753,467 to 2,903,623 bp and the genome coverage ranged from 910× to 994×, with an average of 956×. The GC content of these assemblies ranged from 32.8% to 32.88%. For each genome, the multi-locus sequence type (MLST), *spa* type, and staphylococcal cassette chromosome *mec* (SCC*mec*) type were determined using MLST version 2.0 (https://cge.food.dtu.dk/services/MLST/), spaTyper version 1.0 (https://cge.food.dtu.dk/services/spaTyper/), and SCCmecFinder version 1.2 (https://cge.food.dtu.dk/services/SCCmecFinder/), respectively. Genotypes of KNIH_5618, KNIH_5844, and KNIH_6268 were ST5-t5076-SCC*mec* II, ST72-t664-SCC*mec* IV, and ST89-t375-SCC*mec* II, respectively. The genotypes and assembly statistics are shown in Table 1.

**TABLE 1:** Genotypes and assembly statistics of whole genome sequencing

Isolate	MLST	*spa* type	SCC*mec*	Genome coverage (×)	No. of reads	Genome size (bp)	G + C content (%)	GenBank accession no.	SRA accession no.
KNIH_5618	5	t5076	II	910	250,796	2,903,623	32.88	CP134534	SRR25919446
KNIH_5844	72	t664	IV	994	236,935	2,753,467	32.8	CP134533	SRR25924177
KNIH_6268	89	t375	II	964	260,692	2,862,921	32.8	CP134532	SRR25991521

## Data Availability

Accession numbers are available in Table 1.

## References

[1] Rasmussen RV , Fowler VG , Skov R , Bruun NE . 2011. Future challenges and treatment of Staphylococcus aureus bacteremia with emphasis on MRSA. Future Microbiol 6:43–56. doi:10.2217/fmb.10.155 21162635 PMC3031962

[2] Chong YP , Park S-J , Kim HS , Kim ES , Kim M-N , Park K-H , Kim S-H , Lee S-O , Choi S-H , Jeong J-Y , Woo JH , Kim YS . 2013. Persistent Staphylococcus aureus bacteremia: a prospective analysis of risk factors, outcomes, and microbiologic and genotypic characteristics of isolates. Medicine (Baltimore) 92:98–108. doi:10.1097/MD.0b013e318289ff1e 23429353 PMC4553980

[3] Khatib R , Johnson LB , Fakih MG , Riederer K , Khosrovaneh A , Shamse Tabriz M , Sharma M , Saeed S . 2006. Persistence in Staphylococcus aureus bacteremia: incidence, characteristics of patients and outcome. Scand J Infect Dis 38:7–14. doi:10.1080/00365540500372846 16338832

[4] Clinical and Laboratory Standards Institute . 2020. Performance standards for antimicrobial susceptibility testing. In CLSI document M100, 30th ed. Clinical Laboratory Standards Institute, Wayne, PA.

[5] Patel RK , Jain M . 2012. NGS QC toolkit: a toolkit for quality control of next generation sequencing data. PLoS ONE 7:e30619. doi:10.1371/journal.pone.0030619 22312429 PMC3270013

[6] Ardui S , Ameur A , Vermeesch JR , Hestand MS . 2018. Single molecule real-time (SMRT) sequencing comes of age: applications and utilities for medical diagnostics. Nucleic Acids Res 46:2159–2168. doi:10.1093/nar/gky066 29401301 PMC5861413

[7] Simão FA , Waterhouse RM , Ioannidis P , Kriventseva EV , Zdobnov EM . 2015. BUSCO: assessing genome assembly and annotation completeness with single-copy orthologs. Bioinformatics 31:3210–3212. doi:10.1093/bioinformatics/btv351 26059717

[8] Tatusova T , DiCuccio M , Badretdin A , Chetvernin V , Nawrocki EP , Zaslavsky L , Lomsadze A , Pruitt KD , Borodovsky M , Ostell J . 2016. NCBI prokaryotic genome annotation pipeline. Nucleic Acids Res 44:6614–6624. doi:10.1093/nar/gkw569 27342282 PMC5001611

